# Deep-subwavelength exceptional point in microwave plasmonic resonators for enhanced sensing

**DOI:** 10.1038/s41377-026-02408-0

**Published:** 2026-08-19

**Authors:** Tian Shuo Bai, Xuanru Zhang, Wan Zhu Wang, Jingjing Zhang, Tie Jun Cui

**Affiliations:** 1 State Key Laboratory of Millimeter Waves, Southeast University, Nanjing, 210096 China; 2 School of Information Science and Engineering, Southeast University, Nanjing, 210096 China

**Keywords:** Metamaterials, Nanophotonics and plasmonics

## Abstract

Exceptional points (EPs) in non-Hermitian electromagnetic (EM) systems have been in the spotlight over the past decades due to their remarkable enhancement effects in sensing sensitivity. Here, we explore EPs at a deep-subwavelength scale, where pronounced coupling modulation is achieved via controllable escaping decay channels. This EP state is realized in a pair of microwave plasmonic resonators each with an electrical size of 1/50 wavelength based on the bonding and antibonding eigenmodes. It can concentrate the EM field into an extremely deep-subwavelength mode volume, accompanied by significant field enhancement, hence enhancing the trace-amount sensing capability. A high signal-to-noise ratio is also maintained, owing to the significantly enhanced sensitivity and only modestly increased noise. Experimental validation of the sensing performance is provided by contactless scatterer detection and nanomole-level glucose measurement. The smallest detectable contactless scatterer size is 1/1600 of the wavelength, and the detection limit for glucose reaches 50 nmol in amount of substance at the operating wavelength of 0.32 m. Our results reveal novel modulation mechanism in the deep-subwavelength EM regime, providing a broadened understanding of non-Hermitian EM systems. The nanomole-level microwave sensing experiments envision a new and promising route for label-free biomedical sensing.

## Abstract (web summary)

A microwave plasmonic resonator with exceptional points in deep-subwavelength scale is achieved via controllable escaping decay channels, enabling excellent sensing performance for contactless scatterer detection and nanomole-level glucose measurement.

## Graphical abstract

**Figure:**
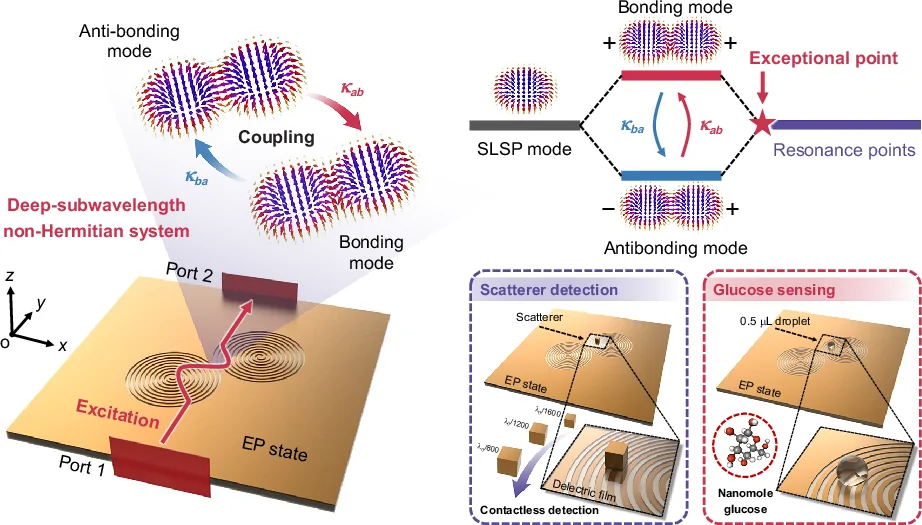

## Introduction

Non-Hermitian electromagnetic (EM) systems are governed by energy exchange with surrounding environment, enabling complex spectra in the corresponding Hamiltonian^1–3^. The non-Hermitian properties can be regulated by precisely controlling gain, loss, and coupling in various physical platforms, such as optics and photonics^4–6^. Remarkably, non-Hermitian systems that obey parity-time (PT) symmetry with balanced gain and loss can support real eigenspectra^7–11^. A unique singularity, known as an exceptional point (EP), occurs at the critical transition between the symmetric and the broken phase, characterized by the degenerate eigenspectra and associated eigenstates. In general, EP phenomena are commonly achieved in fully-passive systems by manipulating the eigenmodes and intermodal interactions, encompassing both propagating^12–14^ and resonant modes^15–19^. EPs can occur across the entire EM spectrum, spanning from optical frequencies^20–23^ to radiofrequency circuits^24–26^. They arise in both electrically large systems and deep-subwavelength structures, and find promising applications for trace-amount sensing^16,27,28^ and feasible wave manipulation^23,29–32^. Considering the broad range of wavelengths and electrical sizes, the EP regulatory mechanisms typically exhibit stringent applicability across diverse domains.

For the optical frequencies, EPs were early demonstrated in optical microcavities in the 2000s, using extra scatterers to modulate coupling coefficients between the clockwise and counterclockwise travelling modes^15^. Then, EPs in microcavities have been extensively realized through gain-loss modulation^33–36^, fully-passive loss engineering^37,38^, optical phase tuning^39–41^, and nonlinear interactions^42^. Optical microcavities serve as an ideal platform for regulating EPs since their electrically large sizes ensure that various tuning mechanisms primarily influence the intermodal coupling coefficient while having minimal impacts on the eigenmodes themselves, resulting in a well-defined and controllable modulation process^43,44^. Leveraging photonic crystals, surface plasmons, and metamaterial structures^45–49^, the optical EPs can be explored at the sizes comparable to or even smaller than the wavelengths^29,50–52^. However, optical manipulations are inherently constrained by size because fabrication precision limits the minimum achievable feature size to only a fraction of the wavelength. From a theoretical perspective, the optical EP regulation requires sufficient size to accumulate adequate phase retardation to tune the intermodal coupling^39,53,54^.

For low-frequency circuits, EP regulation is more convenient due to the availability of diverse passive and active lumped devices, enabling strategical implementation of nonlinear effects^24,55^, non-reciprocal coupling^26,56^, and gain-loss modulation^57,58^ through operational amplifiers, multipliers, and memristors. Beyond their convenience, non-Hermitian circuits enable some unique phenomena that are significantly more challenging to achieve at optical frequencies. For example, nonlinear gain and long-range unidirectional coupling have been reported, overcoming the signal-to-noise ratio (SNR) limitations in traditional optical schemes^24,55,56,58^. Additionally, the schemes of low-frequency non-Hermitian circuits can be implemented in integrated circuit processes, thus promoting more powerful functions with large-scale integration^59^. However, the lumped device model considers the EM process inside the devices only but neglects the distributed phase retardation. This hinders the low-frequency non-Hermitian circuits from dielectric sensing in near-field regions^60,61^.

Microwave EPs at deep-subwavelength scales are located at an intermediate EM regime, where their electrical sizes reside within the quasi-static range, yet wave dynamics remain significant. From a theoretical perspective, the optical and circuit regulation schemes are incompatible with such deep-subwavelength microwave EPs. The downward advancement of the optical method necessitates a large size for accumulating enough phase retardation to attain adequate modulation capability. Several reported microwave EP resonators were regulated using similar extra scatterers as those in optical microcavities and exhibited large electrical sizes^62,63^. The upward advancement of circuit regulation is challenging due to the complexities introduced by the parasitic effects as the frequency increases. From an application perspective, these intermediate EP states combine the sensing enhancement effects of wavelength compression with non-Hermitian EP effects, thereby overcoming the microwave sensing detection limit imposed by their long wavelengths^61^. Meanwhile, they maintain excellent environmental adaption, and economical cost of low-frequency microwave circuits, hence exhibiting promising potentials in ultracompact and portable medical diagnosis systems^64–67^. Hence, it is urgent to propose a principle to modulate the phase and regulate EPs at the deep-subwavelength scale.

**Fig. 1:**
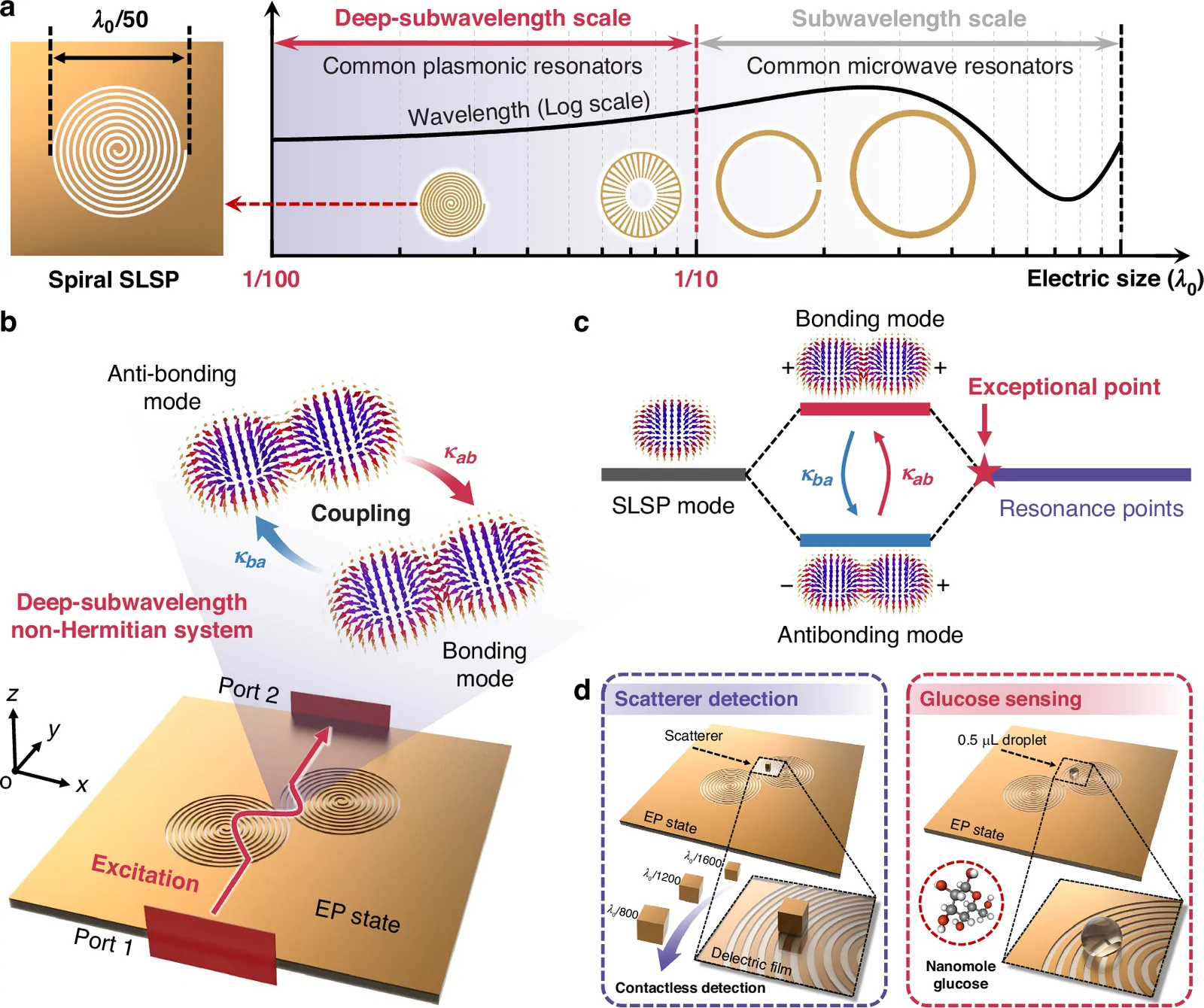
Conceptual illustration of the deep-subwavelength EP state and its enhanced sensing performance. **a** Schematic of the spiral SLSP resonance unit and its electrical size. The electrical size axis and the wavelength diagram are presented on a logarithmic scale. **b** Principle of mode coupling in the coupled-SLSP system. **c** The energy-level diagram describing the antibonding and bonding modes in the coupled-SLSP system. **d** Schematic of contactless scatterer detection and nanomole glucose sensing using the EP state

Here, we propose to construct a deep-subwavelength EP state by two spoof localized surface plasmon (SLSP) units with diameters of λ_0_/50 (where λ_0_ is the operating wavelength), as shown in Fig. 1. The EP state is induced by strong coupling between the antibonding and bonding eigenmodes, while pronounced coupling modulation is enabled through the controllable escaping decay channels of both modes into the excitation microstrip line. The non-reciprocal coherent coupling mechanism is guaranteed by the relative phase difference between antibonding and bonding eigenmodes. The EP state can concentrate EM fields into an extremely deep-subwavelength volume of π/10^6^ λ_0_^3^, accompanied by significant field enhancement, hence enhancing the trace-amount sensing capability. This EP state also maintains a higher SNR compared to the non-EP counterparts, owing to its significantly enhanced sensitivity and modestly increased intrinsic noise. The measured detection limit of contactless scatterers can reach λ_0_/1600, while the detection limit for glucose reaches 50 nmol using the working wavelength of 0.32 m. Our results reveal novel modulation mechanism in the deep-subwavelength intermediate EM regime and deepen the understanding of EM non-Hermitian systems. The microwave sensing experiments demonstrate a nanomole-level detection limit enabled by cheap printed circuit board (PCB) compatible designs, establishing a promising platform for label-free biosensing applications. Additionally, the microwave resonance sensing approach provides excellent environmental stability and high spectral resolution, ensuring that the proposed EP sensor maintains an excellent detectability for frequency splitting.

## Results

### Deep-subwavelength EP state

The SLSP resonance unit is etched in the copper layer using the PCB technology, as shown in Fig. 1a. The spiral arm is 0.15 mm wide and follows the Archimedean spiral equations of *x* = *r·t·*cos(*t*) and *y* = *r·t·*sin(*t*), in which *r* = 0.05 and *t* varies from π to 20π. The spiral structure induces parallel multiple inter-turn capacitances and serial inductances along the metal spiral wire, resulting in a high equivalent capacitance and inductance^68^, which enables EM resonance at physical dimensions far smaller than the operating wavelength. The diameter of the SLSP unit is 6.27 mm, indicating an electric size of λ_0_/50. The dielectric substrate is F4B with a thickness of 0.5 mm, a dielectric constant of 2.65, and a loss tangent of 0.001. The copper thickness is 35 μm. The coupled-SLSP system is composed of two identical SLSP units with a distance *d* of 0.1 mm, and excited by a microstrip line on the backside of the dielectric substrate.

A 2×2 Hamiltonian matrix is used to analyze the eigenmodes in the coupled-SLSP system, which correspond to the antibonding mode (denoted by subscript *a*) and bonding mode (denoted by subscript *b*). The non-Hermitian Hamiltonian is written as:

1 $$\hat{H}=\left(\begin{array}{cc}{\omega }_{a}+i{\gamma }_{a} & {\kappa }_{ab}\\ {\kappa }_{ba} & {\omega }_{b}+i{\gamma }_{b}\end{array}\right)$$

where *ω*_*a*_ (*ω*_*b*_) represents the resonance frequency and *γ*_*a*_ (*γ*_*b*_) represents the damping rate of the antibonding (bonding) mode, and *κ*_*ab*_ and *κ*_*ba*_ denote the intermodal coupling. Enhanced-sensing sensitivity can be realized with a more asymmetric Hamiltonian, which can be accomplished by stronger coupling between the eigenmodes^53,61,63^. The Hamiltonian asymmetries in non-Hermitian resonance systems are indexed as the normalized frequency detuning Δ*ω*/*ω*_0_, where Δ*ω* = *ω*_*b*_ − *ω*_*a*_ and *ω*_0_ = (*ω*_*a*_ + *ω*_*b*_)/2 in the coupled-SLSP system. Figure 1b, c describe the principle of mode coupling and energy-level diagram in the coupled-SLSP system, respectively. The field distributions in Fig. 1b, c of the antibonding and bonding modes are calculated by the eigenmode solver of CST Microwave Studio. Compared to the reported non-Hermitian EP states in both microwave and optical frequencies, this scheme features a larger frequency detuning (Table S1).

**Fig. 2:**
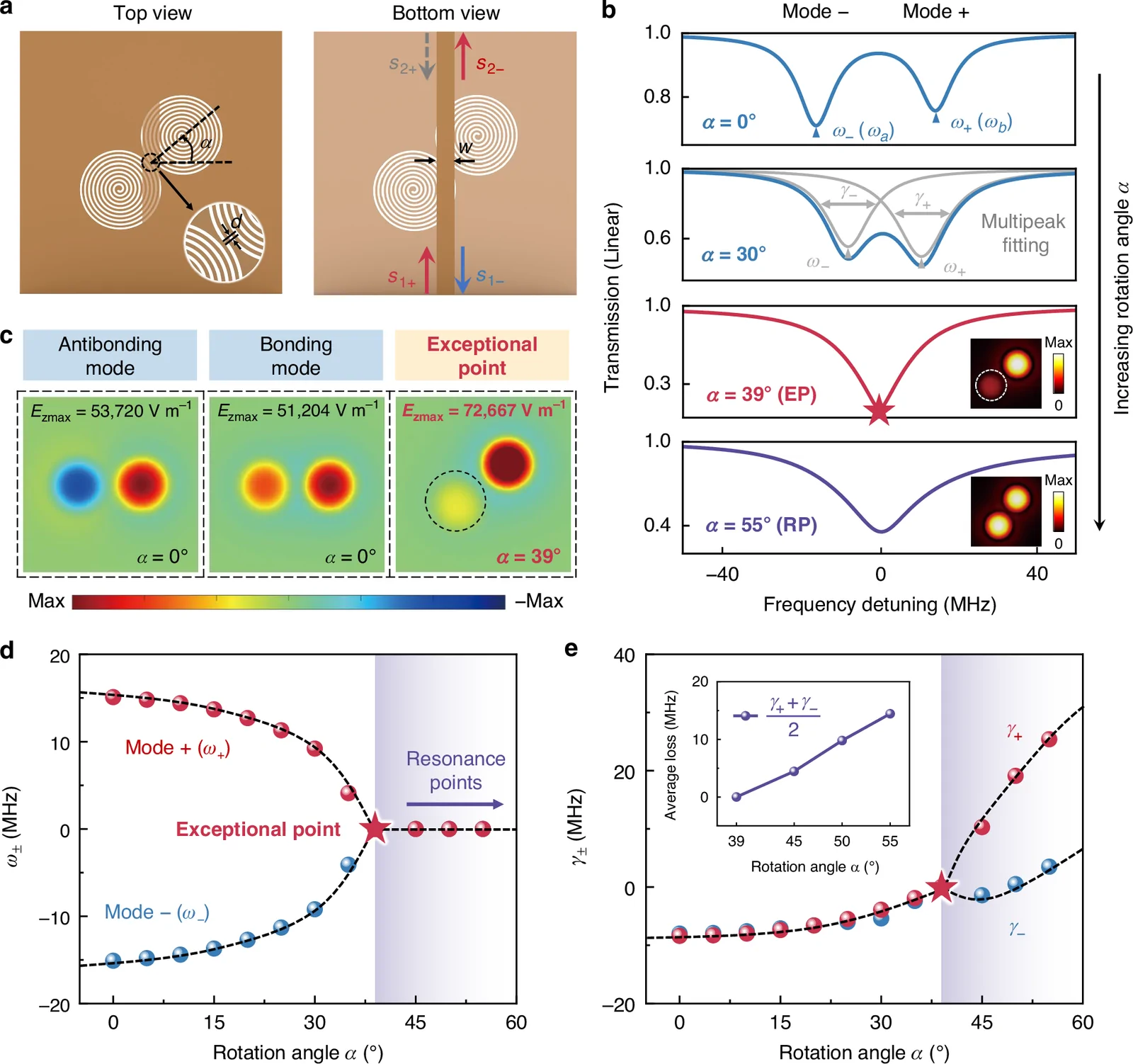
Regulating process of the deep-subwavelength EP state in the coupled-SLSP system. **a** Layout of the coupled-SLSP system, which is excited by the microstrip line on the backside. **b** Evolution of the transmission spectra as the rotation angle *α* increases. The insets show the |*E*_*z*_| distributions at the corresponding resonance states. **c** Electric field *E*_z_ at resonance frequencies for *α* = 0° and *α* = 39° cases. **d**, **e** The real parts *ω*_±_ (**d**) and the imaginary parts *γ*_±_ (**e**) of the complex resonance frequencies at modes ±, varying with the rotation angle *α*. The inset in (**e**) presents the average loss for the RP states

The EP state is induced by rotating the coupled-SLSP system, as shown in Fig. 2a. The excited microstrip line is 1.34 mm wide, with an impedance of 50 Ω. The microstrip line on the backside carries a traveling wave *s*_1+_ towards the coupled-SLSP system from port 1, while there is no input from port 2 (*s*_2+_ = 0). The angle *α*, which is the angle between the microstrip line and the centerline of the coupled-SLSP system, is rotated to adjust the coupling between the antibonding and bonding modes, ultimately approaching the EP state. Figure 2b shows the evolution of the transmission spectra with varying *α*, calculated by the frequency domain solver of CST Microwave Studio. Two separated resonances can be clearly observed in the transmission spectrum at *α* = 0°. As the rotation angle *α* increases, the two resonances gradually converge, eventually coalescing at *α* = 39° and indicating the generation of the EP state. As *α* increases further, the resonance frequency keeps coalescing while the resonance bandwidth gradually broadens, corresponding to the resonance point states (RPs)^69^. The EP state (*α* = 39°) exhibits a quality factor (Q-factor) of 49, which is higher than the value of 31 of the RP state (*α* = 55°).

To analyze the EP regulating process, a multipeak Lorentz fitting method is adopted to fit the transmission spectra of the overlapped resonances (Fig. 2b and Multipeak fitting method in Materials and Methods). The resonance frequencies *ω*_*+*_ (*ω*_−_) and the damping rates *γ*_*+*_ (*γ*_−_) of the two evolving peaks (denoted by subscripts + and −, respectively) are demonstrated in Fig. 2d, e We make a distinction between the + ( − ) modes and the *a* (*b*) modes, in which the *a* (*b*) modes are intrinsic eigenmodes with fixed resonance frequencies and damping rates, while the + ( − ) modes correspond to two peaks observed in the transmission spectra whose resonance frequencies and damping rates vary with *α*. Before reaching the EP state (when *α* < 39°), *ω*_*+*_ and *ω*_−_ are completely separated, while *γ*_*+*_ and *γ*_*−*_ are approximately equal (Note S1). Both *ω*_*+*(*−*)_ and *γ*_*+*(*−*)_ coalesce at *α* = 39° simultaneously, indicating the occurrence of the EP state, as marked by the red pentagram in Fig. 2b,d,e. The RP states (when *α* > 39°) manifest as degenerate real parts *ω*_*+*(*−*)_ with split imaginary parts *γ*_*+*(*−*)_, as shown in Fig. 2d, e. The increasing damping rates confirm the mechanism behind the lower Q-factor of the RP states (inset in Fig. 2e).

When *α* = 0°, the two split resonances (modes + and −) are exactly the antibonding and bonding modes (modes *a* and *b*), as shown in Fig. 2c, since the intermodal coupling is weak in this symmetric case (“Theoretical analysis of the deep-subwavelength EP state” section). For the EP state when *α* = 39°, the electric field is concentrated in one SLSP unit, while the electric field in the other one is always suppressed, as shown in insets of Fig. 2b, c. For the RP state, the electric field is alternatively concentrated in each SLSP unit in each half period, as shown in the insets of Fig. 2b. The transient electric field evolution of all modes discussed here is presented in Fig. S1. Comparing |*E*_*z*_| distributions of the EP and RP states in the insets of Fig. 2b, these measurable field distributions provide a practical method to distinguish the EP and RP states in experiments. In fact, the EP state enables enhanced wave-matter interaction, attributed to its strong EM concentration in the deep-subwavelength regime, which is quantified by the field enhancement and mode volume. As shown in Fig. S2, the EP state features a 1.85-fold electric field enhancement than the microwave plasmonic resonator, and a 5.81-fold enhancement than the conventional microstrip ring resonator (MRR). Meanwhile, the EP state features a lateral size of λ_0_/50, and half vertical size of λ_0_/200, as calculated by evanescent field distance, leading to an extremely deep-subwavelength mode volume of π/10^6^ λ_0_^3^. These properties make the proposed EP state promising for enhancing trace-amount sensing capabilities and nonlinear effects^70,71^.

### Theoretical analysis of the deep-subwavelength EP state

In this section, we theoretically elucidate the evolution process of the deep-subwavelength EP state using the temporal coupled-mode theory (TCMT)^72,73^. The two modes we used to construct the EP state are antibonding and bonding modes, as discussed above. We highlight the challenge in regulating the deep-subwavelength EP state, as it requires pronounced coupling modulation capacity in an extremely small electrical size. Here, the EP state is induced by the strong coupling between the antibonding and the bonding eigenmodes. The controllable escaping decay of both modes facilitates pronounced coupling modulation capacity, which is implemented through rotating the coupled-SLSP system. The theory ignores the intrinsic loss (material absorption loss and radiation loss) in either the antibonding or bonding mode, since such losses do not contribute to the intermodal coupling or the EP state induction (Note S2).

The energy in such an approximately closed system (assuming no external gain or intrinsic loss) is conserved. Hence, the finite amplitude of the antibonding (bonding) mode within the coupled-SLSP system can decay through the escaping decay *γ*_*ea*_ (*γ*_*eb*_), transferring energy to the two physical ports via microstrip line. The antibonding and bonding modes can strongly couple via the same escaping decay channel (refer to microstrip line), and the escaping decay governs the coupling modulation. The decay-induced coupling is analogous to dissipative coupling via the free-space radiative channel, occurring between radiative modes with identical propagation phases^74^. In this non-radiating coupled-SLSP system, the decay-induced coupling features non-reciprocal coherent coupling, which is guaranteed by the π/2 relative phase difference between the antibonding and bonding modes (Coupling mechanism in “Materials and methods” section). Considering the energy conservation rule and the specific phase relationship between the antibonding and bonding modes, we derived the coupling coefficient *κ*_*ab*_ (*κ*_*ba*_) between the two modes (Coupling mechanism in “Materials and methods” section):

2 $${\kappa }_{ab}=-{\kappa }_{ba}=-\sqrt{{\gamma }_{ea}{\gamma }_{eb}}$$

Equation (2) elucidates that the coupling coefficients *κ*_*ab*_ and *κ*_*ba*_ are non-reciprocal, exhibiting coherent constructive (positive real number) and coherent destructive (negative real number) coupling of internal eigenmodes. Then, the controllable escaping decay on intermodal coupling is analyzed by rotating the single SLSP resonance unit, as demonstrated in Fig. S3. During the rotation dynamics, the excitation microstrip line and the resonance unit encounter stronger coupling, resulting in a significant increase in escaping decay. Hence, rotating the coupled-SLSP system facilitates an increase in the escaping decay of both the antibonding and bonding modes as *α* increases from the *α* = 0° case, which in turn enhances the intermodal coupling between the two modes, following the relation $|{\kappa }_{ab}|=\sqrt{{\gamma }_{ea}{\gamma }_{eb}}\propto \alpha$.

In actual systems, intrinsic losses should be considered, even with high Q-factor resonance. The intermodal coupling in the coupled-SLSP system is described with a TCMT equation:

3 $$i\left(\begin{array}{c}\omega A\\ \omega B\end{array}\right)=i\left(\begin{array}{cc}{\omega }_{a}+i{\gamma }_{a} & {\kappa }_{ab}\\ {\kappa }_{ba} & {\omega }_{b}+i{\gamma }_{b}\end{array}\right)\left(\begin{array}{c}A\\ B\end{array}\right)=i\hat{H}\left(\begin{array}{c}A\\ B\end{array}\right)$$

where the amplitudes are denoted as *A*(*x*, *y*, *z*) for the antibonding mode and *B*(*x*, *y*, *z*) for the bonding mode, which are two positive real functions that depend solely on the spatial EM distributions. $\hat{H}$ is the non-Hermitian Hamiltonian. The damping rates *γ*_*a*_ (*γ*_*b*_) of the modes include both the intrinsic loss *γ*_0*a*_ (*γ*_0*b*_) and the escaping decay *γ*_*ea*_ (*γ*_*eb*_) for the antibonding (bonding) mode. Here, the intermodal coupling depends solely on the escaping decay *γ*_*ea*_ (*γ*_*eb*_), and variations in intrinsic loss *γ*_0*a*_ (*γ*_0*b*_) of the modes fail to achieve the expected EP state (Note S2). The eigenfrequencies *ω*_±_ in this non-Hermitian system follow:

4 $${\omega }_{\pm }={\omega }_{0}+i{\gamma }_{0}\pm \sqrt{{(\Delta \omega +i\Delta \gamma )}^{2}+{\kappa }_{ab}{\kappa }_{ba}}={\omega }_{0}+i{\gamma }_{0}\pm \sqrt{{(\Delta \omega +i\Delta \gamma )}^{2}-{\gamma }_{ea}{\gamma }_{eb}}$$

where *ω*_0_ = (*ω*_*a*_ + *ω*_*b*_)/2, *γ*_0_ = (*γ*_*a*_ + *γ*_*b*_)/2, *Δω* = (*ω*_*a*_ − *ω*_*b*_)/2, and Δ*γ* = (*γ*_*a*_ − *γ*_*b*_)/2. The antibonding and bonding modes exhibit approximately the same damping rates (Δ*γ* = 0), as demonstrated in Note S1. The two modes exhibit identical intrinsic losses, dominated by absorption due to negligible radiation losses in this deep-subwavelength system. Meanwhile, the escaping decays of two modes remain identical due to the system’s reciprocity. The deep-subwavelength EP state can be achieved when the square root term approaches zero i.e., Δ*ω*^2^ = *γ*_*ea*_*γ*_*eb*_ (*Δω* = 15.1 MHz and$\sqrt{{\gamma }_{ea}{\gamma }_{eb}}$= 15 MHz), exhibiting a single resonance as the eigenfrequencies coalesce. For the *α* = 0° case, the frequency detuning Δ*ω* greatly exceeds $\sqrt{{\gamma }_{ea}{\gamma }_{eb}}$ (Δ*ω* = 15.1 MHz and$\sqrt{{\gamma }_{ea}{\gamma }_{eb}}$= 2 MHz) due to the weak escaping decay in this symmetrical configuration, and a clear resonance splitting is demonstrated in Fig. 2b. A larger $\sqrt{{\gamma }_{ea}{\gamma }_{eb}}$ than the frequency detuning Δ*ω* (Δ*ω* = 15.1 MHz and$\sqrt{{\gamma }_{ea}{\gamma }_{eb}}$> 15 MHz) also results in a single resonance distinct from the EP state, due to the eigenfrequencies manifest as degenerate real parts with split imaginary parts in the *α* > 39° case. The transmission spectra calculated by the TCMT model coincide well with the full-wave EM simulation results, as shown in Note S3, which conforms to the structure-induced escaping decay mechanism underlying the proposed deep-subwavelength EP state. The positive correlation relation |*κ*_*ab*_ | ∝ *α* is also validated.

### Sensitivity analysis of the deep-subwavelength EP state

**Fig. 3:**
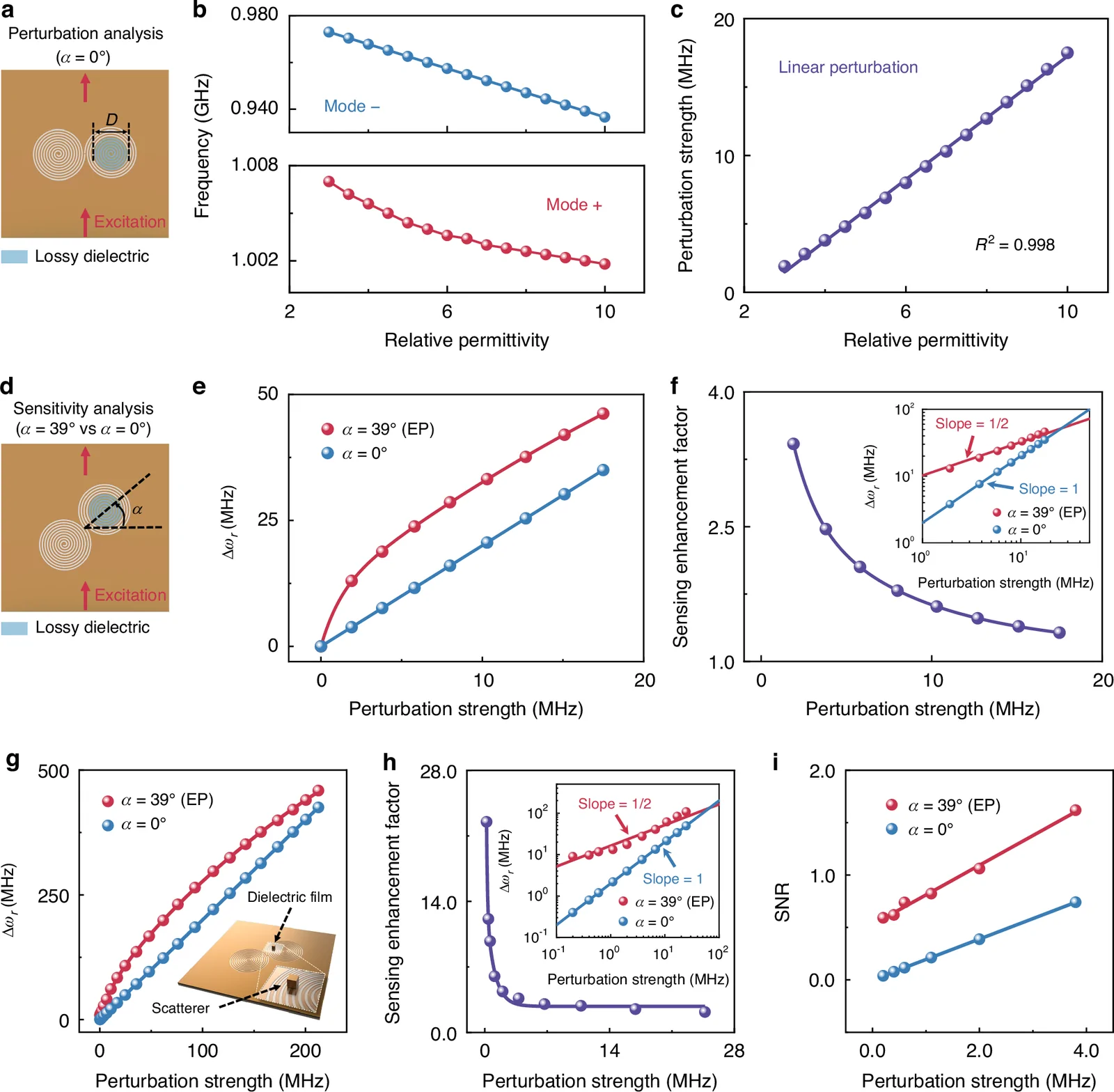
Sensitivity analysis of the deep-subwavelength EP state. **a** The coupled-SLSP system is covered by a lossy dielectric film when *α* = 0°. **b** The real frequencies of mode ± varying with the relative permittivity under test. **c** The perturbation strength varies with the relative permittivity under test, along with their linear fitting result. **d** The EP state (*α* = 39°) covered by a lossy dielectric film under test. **e** Relative frequency splitting of the EP state (*α* = 39°) and the *α* = 0° case varying with the perturbation strength. **f** The sensing enhancement factor comparing the EP state with the *α* = 0° case in lossy dielectric sensing. The inset demonstrates a logarithmic plot of the relative frequency splitting versus the perturbation strength. **g** Relative frequency splitting of the EP and the *α* = 0° state responding to the contactless scatterer size. The inset shows the contactless scatterer detection. **h** The sensing enhancement factor comparing the EP state and the *α* = 0° state as functions of the perturbation strength in contactless scatterer detection. The inset shows a logarithmic plot of the relative frequency splitting curves. **i** Estimated SNR comparing the EP state and the *α* = 0° state as a function of perturbation strength in contactless scatterer detection

Two sensing scenarios are considered to evaluate the sensing performance by full-wave simulations: lossy dielectric sensing (corresponding to solution concentration sensing in experiments) and contactless scatterer sensing. The dielectric under test is assigned a loss tangent of 0.025 to mimic the loss characteristics of aqueous solutions. The lossy dielectric is positioned on top of one SLSP resonance unit, as shown in Fig. 3a, d. The diameter *D* of the lossy dielectric is set to 4 mm, with a thickness of 0.1 mm. The sensing performance of the EP state is compared with the case of *α* = 0° to evaluate its enhancement effect, since there is no diabolic point with degenerate eigenfrequencies and orthogonal eigenstates in the coupled-SLSP system. The *α* = 0° case corresponds to a splitting state with weak intermodal coupling between the antibonding and bonding eigenmodes, and can therefore be approximated by a diagonalizable Hamiltonian with orthogonal eigenstates. A relative frequency splitting is defined as Δ*ω*_*r*_ = Δ*ω* − Δ*ω*_*original*_ to standardize the frequency splitting variance caused by the perturbation, where the EP and *α* = 0° case in the absence of perturbation feature different original frequency splittings Δ*ω*_*original*_. As demonstrated in Fig. 2b, d, Δ*ω*_*original*_ is 30.2 MHz for the *α* = 0° case, while Δ*ω*_*original*_ is 0 MHz for *α* = 39° due to *ω*_*+*(*−*)_ coalesce at the EP state. Meanwhile, Δ*ω* represents the ultimate frequency splitting, fitted by the multiple fitting method (Materials and methods) in the presence of perturbation (i.e., with dielectric under test or scatterers).

For the dielectric sensing scenario, the frequencies of the + ( − ) modes in the *α* = 0° case are simulated, as shown in Fig. 3b. Figure 3c validates the linear relationship between the perturbation strength and the relative permittivity of the dielectric under test. The perturbation strength is defined as half of the relative frequency splitting of the *α* = 0° case^15^. The R-square (coefficient of determination) equals 0.998, which indicates a good linear fit. The dielectric sensing performance of the EP state is simulated as shown in Fig. 3d–f, compared with the *α* = 0° case. The EP state exhibits superior relative frequency splitting than the *α* = 0° case. The sensing enhancement factor is defined as the ratio of relative frequency splitting Δ*ω*_*r*_ at the EP state (*α* = 39°) to that at the *α* = 0° state. The sensing enhancement factor reaches a maximum under extremely small perturbation strength, and exponentially decreases as the perturbation strength increases. The inset of Fig. 3f shows the relationship between the relative frequency splitting and perturbation strength in a logarithmic plot. The EP state presents a slope of 1/2 of that of the *α* = 0° case, confirming the square-root response to the perturbation strength in the vicinity of the second-order EP state. The simulated results for the contactless scatterer detection scenario are shown in Fig. 3g, h. The scatterers and the EP sensor are separated by a dielectric film to achieve contactless (non-conductive) sensing (inset in Fig. 3g). The dielectric film features a dielectric constant of 3.5, a thickness of 0.02 mm, and a size of 3 mm × 3 mm. Similar to the dielectric sensing case, the EP state exhibits significantly enhanced sensitivity compared with the *α* = 0° case, especially when the scatterer size is small. The logarithmic plot of the relative frequency splitting curves in the inset of Fig. 3h also confirms the square-root response of the second-order EP state.

These results have demonstrated the enhanced frequency splitting sensitivity by constructing the deep-subwavelength EP-based sensor. However, the noise in the vicinity of EP states is increased compared to the non-EP counterparts, which are derived from the nonorthogonality of eigenmodes^26^. The noise leads to a broader bandwidth in transmission spectra and then enhances the random frequency fluctuations. Consequently, the precision in actual measurements may not be improved, even though the frequency splitting is indeed enhanced in the vicinity of an EP state^75–77^. The actual benefits of sensors could be quantified using Fisher information within a parameter-estimation framework^78–80^. For EM resonance sensing, the minimum detectable frequency shift in the presence of noise is primarily governed by the resonance bandwidth, as quantified using Fisher information^81^. The random frequency fluctuations induced by both broad- and narrow-bandwidth resonance are similar and therefore do not increase the minimum detectable frequency shift when the measuring equipment features high spectral resolution, such as a vector network analyzer (VNA) for measuring microwave transmission and reflection spectra, as compared in Fig. S16a, b. Hence, an estimated SNR is defined as 2Δ*ω*_*r*_/(*γ*_*+*_ *+ γ*_*−*_), which quantifies the enhancement of frequency splitting relative to the bandwidth increase^55,63^. Here, Δ*ω*_*r*_ represents the frequency splitting variation caused by perturbation, which can evaluate the magnitude of the detectable sensing signal, and (*γ*_*+*_ *+ γ*_*−*_)/2 represents the average loss of the two overlapping resonances. However, the minimum detectable frequency shift is governed by both the resonance bandwidth and the random frequency fluctuation when the measuring equipment features a low spectral resolution. The frequency fluctuation induced by the broad-bandwidth resonance remains more significant than that of the narrow-bandwidth resonance, as compared in Fig. S16c, d. Figure 3i shows the estimated SNR comparing the EP state with the *α* = 0° state as a function of perturbation strength, where the fitted sensing signal Δ*ω*_*r*_ and average loss (*γ*_*+*_ *+ γ*_*−*_)/2 are shown in Fig. S4. The SNR in both cases increases with the perturbation strength, while the EP state persistently maintains a higher SNR owing to its significantly enhanced sensing enhancement factor and modest growth in noise.

As shown in Fig. 3f, the maximum sensing enhancement of the EP state compared with the *α* = 0° case reaches 3.4 when the perturbation strength equals 1.9 MHz for the dielectric sensing scenario. This enhancement value evaluates the EP effect on the basis of deep-subwavelength SLSP resonance. We remark that the deep-subwavelength mode confinement in the SLSP unit also enhances the wave-matter interactions^66^, leading to a sensing enhancement of 36-fold compared to conventional MRR resonating at the same frequency (considering a cylinder-shaped dielectric under test with a diameter of 4 mm and a thickness of 0.1 mm). Meanwhile, the proposed SLSP resonator features a smaller footprint than the MRR, which broadens its application potential in large-scale sensor integration. Consequently, the combination of the deep-subwavelength effect and the non-Hermitian EP effect results in a 62-fold enhancement compared to the MRR. The detailed comparison of the sensing sensitivity is demonstrated in Fig. S5.

### Sensing validations

**Fig. 4:**
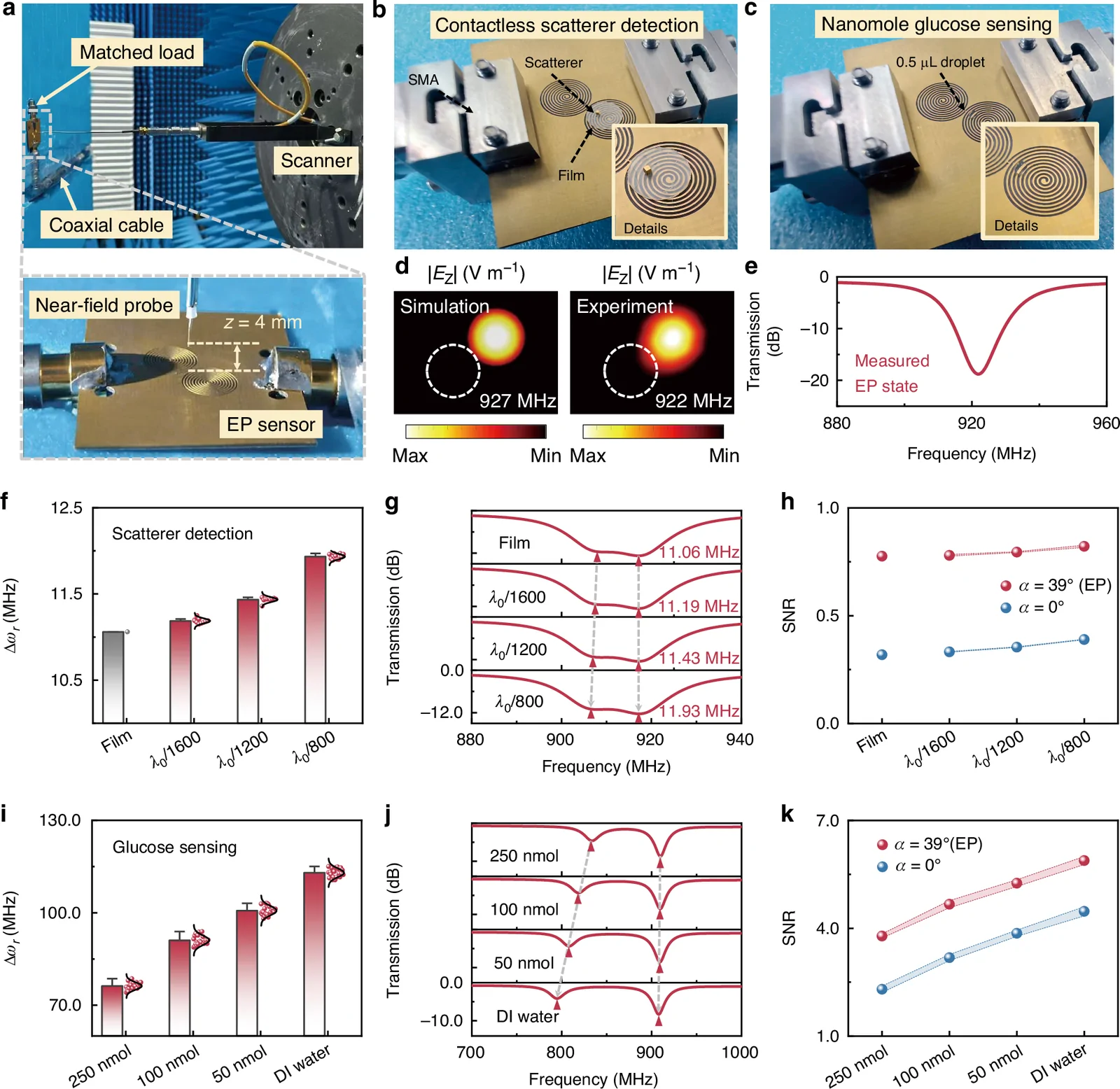
Experimental validation of the sensing performance. Experimental setup photographs for near-field mapping in (**a**) contactless scatterer detection in (**b**), and trace-amount glucose sensing in (**c**). **d** Simulated and measured |*E*_*z*_| distributions of the EP state. **e** Measured transmission spectrum of the EP state. **f** Frequency splitting of the EP state responding to the sealing film, and the scatterers of different sizes above the film. **g** Measured spectra for detecting contactless scatterers of different sizes. **h** Measured SNR comparing the EP state and the *α* = 0° state for contactless scatterers detection. The dashed lines and colored shadows represent the standard deviation for measured SNR. **i** Frequency splitting of the EP state responding to DI water, and the glucose solution of various concentrations. **j** Measured spectra for detecting glucose solution of various concentrations with a VUT of 0.5 μL. **k** Measured SNR comparing the EP state and the *α* = 0° state for glucose sensing. The dashed lines and colored shadows represent the standard deviation for measured SNR

The actual benefits of the EP state are experimentally demonstrated by its significantly enhanced sensitivity and only modestly increased noise compared to the non-EP counterparts, as shown in Fig. 4 and supported by statistical analysis in contactless scatterer detection and trace-amount glucose sensing. Experimental measurement variations are derived from random frequency fluctuations induced by bandwidth, frequency self-drift of the VNA, and experimental operation errors across multiple measurements. Notably, the measurement variations using the EP state are similar to the non-EP states operated at the identical experimental configuration. Even though the EP state exhibits a broad bandwidth, the high spectral resolution of the VNA ensures that random frequency fluctuations remain limited, as demonstrated in Fig. S16a, b. Consequently, the proposed EP sensor can still achieve the excellent experimental detection limits, as shown in Fig. 4 and Table S2. Photographs of the whole experimental setup are shown in Fig. S6. Before conducting the sensing experiments, the |*E*_*z*_| distribution on top of the EP sensor is measured. The photographs of the near-fielding mapping system and the fabricated sample under test are shown in Fig. 4a, while the details of the setup are referred to Note S4. Notably, the distance between the near-field probe and the surface of the EP sensor was set to 4 mm, according to the simulated evanescent field characteristics of the EP sensor, as shown in Fig. S15. This ensures that the near-field probe causes minimal perturbation to the EM field of the EP state. The measured |*E*_*z*_| distribution in Fig. 4d perfectly resembles the simulated result, and confirms that the fabricated sample is located indeed at the EP state. We remark that the RP states also present a single spectral peak, but their |*E*_*z*_| fields are distributed across both SLSP units. Therefore, the experimental distinction between the RP and EP states is only possible through the near-field mapping. Fig. 4e shows the measured transmission spectrum of the deep-subwavelength EP state. The measured EP resonance frequency is 922 MHz with a Q-factor of 55. The higher Q-factor compared with the simulated one can be attributed to lower resonance intensity in the experiments^61^. In microwave resonance sensing, excellent frequency stability can enhance the trace-amount detection capacity during long-term monitoring and dynamic measurements. The fabricated EP sensor demonstrates a slight frequency drift by monitoring the transmission spectra over an extended period, as shown in Fig. S7. Additionally, the fabricated EP sensor has excellent thermal stability against the temperature increases induced by the substrate heating and metal ohmic loss, as shown by the transmission spectra measured at 23 °C and 100 °C (Fig. S8). These properties confirm the robustness of the proposed EP sensor for a wide range of sensing applications.

In the contactless scatterer detection, a circular sealing film (Parafilm, Amcor) with a diameter of 4 mm and a thickness of 127 μm is covered over the SLSP resonance unit to separate the EP sensor and the scatterer. The ultrathin profile and small contact area of the sealing film ensure minimal perturbation to the EP sensor. Then, copper cubes with a width of 0.2 mm (λ_0_/1600), 0.3 mm (λ_0_/1100), and 0.4 mm (λ_0_/800) are placed on the sealing film, as shown in Fig. 4b. The measured frequency splitting of the EP state is shown in Fig. 4f, responding to the sealing film, and the scatterers of different sizes above the film. The mean, standard deviation, and normal distribution are obtained from ten independently repeated measurements. For each measurement, the scatterer is placed at a fixed position above the film. Due to the slight randomness in both the position and orientation of the scatterer, the frequency splitting shows slight variations across multiple measurements. However, as the electrical size of the scatterer increases, the frequency splitting features progressively increasement. The EP sensor also has the potential to perform scatterer counting (Fig. S9) and detect deeper subwavelength targets. The measured spectra are presented in Fig. 4g. A frequency splitting of 11.06 MHz occurs when the film is placed on the surface without scatterers. Larger scatterers result in greater frequency splitting, which confirms the consistency between the experimental and simulation results. The measured detection limit reaches λ_0_/1600, where Δ*ω* = 11.19 MHz. Compared to other reported EP sensors across both microwave and optical frequencies, the proposed EP sensor demonstrates a significantly lower detection limit for scatterer detection, as shown in Table S2. The sensing performance of the *α* = 0° state under identical experimental conditions is given in Fig. S9 for comparison, demonstrating smaller frequency splitting, which coincides well with the simulated results in Fig. 3g.

The measured SNR is calculated from the measured transmission spectra, according to the definition of estimated SNR mentioned above. The standard deviations of the measured SNR across multiple measurements are shown in the dashed lines and colored shadows of Fig. 4h, proving better consistency. The EP state shows a higher SNR, which coincides well with the simulated results in Fig. 3i. The measured SNR in the EP state is less than 1, indicating that the defined noise exceeds the sensing signal when detecting small perturbations. However, the measured derivations (Fig. 4f) are much smaller than the theoretically defined noise (averaged resonance widths). The microwave resonance sensing approach provides excellent environmental stability and high spectral resolution, ensuring that the microwave sensors subjected to broad bandwidths can still achieve an excellent detectability for frequency splitting (Note S5). Hence, the SNR issue in EP sensing is less critical compared to optical and terahertz sensing technologies^61,66,67^. Both the high SNR value and the high measurement resolution ensure the superior performance of the proposed EP sensor.

In trace-amount glucose sensing, the glucose solution is manipulated using a microinjector, and placed onto the EP sensor surface, as shown in Fig. 4c. The volume under test (VUT) is fixed to 0.5 μL with an electrical size of 1.44 × 10^−8^ λ_0_^3^ (the operating wavelength is 0.32 m). The perturbation strength of the glucose solution decreases as its concentration increases, due to the reduction in relative permittivity with higher glucose concentrations^66,82^. The glucose concentrations used are 18, 36, and 90 mg mL^−1^, corresponding to 50, 100, and 250 nmol glucose in the VUT of 0.5 μL. The glucose solution is positioned along the centerline of the coupled-SLSP system. The distance between the glucose solution and the center of one SLSP resonance unit is fixed at 1.5 mm in twenty independently repeated measurements. The water evaporation during trace-amount glucose sensing reduces the perturbation strength on the EP sensor, resulting in a smaller frequency splitting. Hence, the transmission spectra must be recorded rapidly to minimize the effects of evaporation. The mean, standard deviation, and normal distribution are calculated from these measured data. Apparently, the frequency splitting decreases as the glucose concentration increases, as shown in Fig. 4i, j. The reporting limit in amount of substance for glucose measurement is 50 nmol in the experiments, around the resonance wavelength of 0.32 m. The significant size contrast between the wavelength and the sensing target highlights the sensitivity advantage of the deep-subwavelength EP state. The comparison of measured SNRs with standard deviations across multiple measurements in the EP state and *α* = 0° state is shown in Fig. 4k, which also proves better consistency. The measured SNR is higher than that in the scatterer detection scenario, as larger sensing signals are generated when detecting high-dielectric aqueous solutions. The experimental results for glucose sensing of the *α* = 0° state are also given in the Fig. S10. The frequency splitting remains lower than that of the EP state, further confirming the sensing-enhanced capability of the EP state. The EP sensor also enables specific sensing capability in complex biological matrices, such as blood or serum where multiple dielectric components coexist. The EP sensor is primarily interacted with the specific antigens by constructing a layer of uniformly distributed antibodies on the sensor surface. However, non-specific interferences may enhance the perturbation strength to the EP sensor, which potentially leads to false-positive predictions or misdiagnoses of more severe conditions. These interferences can be effectively mitigated by gentle washing steps, owing to their low affinity for non-specific adsorption.

## Discussion

We realized a deep-subwavelength EP state using a pair of coupled-SLSP units, which are only 1/50 of the operating wavelength. The key challenge lies in achieving pronounced coupling modulation capacity at the quasi-static scale, which has been realized through controllable escaping decay channels. Our findings have deepened the understanding of non-Hermitian EM systems at the extremely deep-subwavelength scale. Meanwhile, we realized the nanomole-level molecule sensing using a VUT of 0.5 μL at the microwave operating wavelength of 0.32 m. The detection limit is sensitive enough for various biomedical diagnostic markers, and the EP sensor fabricated using the PCB process offers a feasible method for a compact and portable sensing system with a low economic cost, excellent environmental adaptability, and convenient operation. More efforts to further improve the detection limit should be allocated towards enhancing the SNR (i.e., the Q-factor) of the second-order EP sensor and developing more effective detection methods.

## Materials and methods

### EM simulations

EM simulations were calculated using the frequency domain solver of the CST Microwave Studio with a specified accuracy of 1 e^−6^. The frequency range was set from 0 to 2 GHz, with a total sample point of 10,001. A tetrahedral mesh was used, with a maximum cell size of 1/60 of the wavelength near the model and 1/30 of the wavelength in the background. The maximum mesh step width of the resonance structure is set to 0.15 mm to further refine the mesh.

### Multipeak fitting method

The multipeak fitting method was applied to fit the spectrum, which is modeled as the superposition of two Lorentzian functions. The resonance frequencies are determined by the positions of the two Lorentzian functions, while the damping rates are calculated from the full width at half maximum (FWHM) of the two Lorentzian functions. The determined resonance frequencies and damping rates show good agreement with the cumulative peak fitting results.

### Materials and fabrications

The PCB substrate was F4B with a dielectric constant of 2.65 and a loss tangent of 0.001. The printed metal patterns consisted of 35 μm thick copper, with a 2 μm thick gold layer electroplated on the copper surface as an antioxidant coating. The dielectric film in contactless scatterer detection experiment was using the sealing film (Parafilm, Amcor).

### Measurement setup

Keysight VNA E5063A was used to measure the transmission spectra of the EP sensor. A sweeping range from 870 to 970 MHz with 2001 sweep points was set during the contactless scatterer detection. A sweeping range from 700 to 1000 MHz with 3001 sweep points was set during the trace-amount glucose measurements. The IF bandwidth was set at 15 kHz to lower the noises and jitters.

### Coupling mechanism

The evanescent wave coupling between two SLSP resonance units leads to the energy-level splitting, and generates the antibonding and the bonding modes. The resonance frequency of the single SLSP resonance unit is *ω*_*s*_. The evanescent wave coupling exhibits coherent coupling, with the coupling strength denoted as *κ*_+_ (*κ*_−_), where both *κ*_+_ and *κ*_−_ are purely real numbers. Hence, the resonance frequency is *ω*_*a*_ = *ω*_*s*_ − *κ*_−_ for the antibonding mode, and *ω*_*b*_ = *ω*_*s*_ + *κ*_+_ for the bonding mode. Here, the basic vector can be chosen as the antibonding and bonding modes due to their intrinsic orthogonality. The complex amplitude of two SLSP resonance units (*u*_1_ and *u*_2_), the antibonding mode (*a*) and the bonding mode (*b*) follows:

5 $$\left(\begin{array}{c}a\\ b\end{array}\right)=\left(\begin{array}{cc}{c}_{11} & -{c}_{12}\\ {c}_{21} & {c}_{22}\end{array}\right)\left(\begin{array}{c}{u}_{1}\\ {u}_{2}\end{array}\right)=M\left(\begin{array}{c}{u}_{1}\\ {u}_{2}\end{array}\right)$$

where *c*_*ij*_ (*i*, *j* = 1, 2) represents the linear combination coefficient. Apparently, the rank of matrix *M* is 2, indicating that the antibonding and bonding modes are mutually orthogonal. The coupling mechanism between the antibonding and bonding modes can be explained by analyzing the energy conversion and conservation using the TCMT^83^. According to the TCMT, the dynamic equations for resonance modes *m* are given by:

6 $$\left\{\begin{array}{c}\frac{dm}{dt}=(i\Omega -{\Gamma }_{e})m+{K}^{T}{s}_{+}\\ {s}_{-}=C{s}_{+}+Dm\end{array}\right.$$

where *m* represents the resonance mode (*a b*)^T^ within the coupled-SLSP system, *s*_+_ represents the external excitation waves (*s*_1+_ *s*_2+_)^T^ from two physical ports. *s*_−_ represents the outgoing waves (*s*_1−_ *s*_2−_)^T^ from two physical ports. *Ω* represents resonance frequencies of the modes, *Γ*_*e*_ represents the decay of the modes and the coupling between modes. *K* represents the coupling matrix expressing the degree of coupling between the resonance modes *m* and the external excitation waves *s*_+_. The scattering matrix *C* describes the transmission (*t*) and reflection (*r*) between waves in the coupled-SLSP system, and the coupling matrix *D* describes the coupling between the resonance mode and the physical ports with a certain amplitude and phase. Such a coupled-SLSP system is described by matrixes:

7 $$\begin{array}{c}\Omega =\left(\begin{array}{cc}{\omega }_{a} & 0\\ 0 & {\omega }_{b}\end{array}\right)\,{\Gamma }_{e}=\left(\begin{array}{cc}{\gamma }_{ea} & {\gamma }_{c}\\ {{\gamma }_{c}}^{\ast } & {\gamma }_{eb}\end{array}\right)\\ C=\left(\begin{array}{cc}r & t\\ t & r\end{array}\right)\,D=\left(\begin{array}{cc}{d}_{11} & {d}_{12}\\ {d}_{21} & {d}_{22}\end{array}\right)=\left(\begin{array}{cc}|{d}_{11}|{e}^{i{\varphi }_{11}} & |{d}_{12}|{e}^{i{\varphi }_{12}}\\ |{d}_{21}|{e}^{i{\varphi }_{21}} & |{d}_{22}|{e}^{i{\varphi }_{22}}\end{array}\right)\end{array}$$

where *γ*_*c*_ represents the mode coupling generated by the escaping decay via the microstrip circuits.

With the introduction of external excitation waves *s*_+_, the resonance mode *m* reaches a steady state, where the amplitude and energy remain constant over time. According to the conservation of energy, the resonance mode will decay exponentially into all the ports when the external excitation *s*_+_ is absent. The energy of the resonance modes *m* in the coupled-SLSP system thus varies as:

8 $$\begin{array}{c}\frac{d({m}^{+}m)}{dt}=\frac{d({m}^{+})}{dt}m+{m}^{+}\frac{d(m)}{dt}\\ ={m}^{+}(-i{\varOmega }^{+}-{{\varGamma }_{e}}^{+})m+{m}^{+}(i\varOmega -{\varGamma }_{e})m\\ =-2{m}^{+}{\varGamma }_{e}m\end{array}$$

here, we note that *m*^+^ is the Hermitian adjoint of the resonance amplitude, and both *Ω* and *Γ*_*e*_ are Hermitian matrixes (*Ω*^*+*^ = *Ω* and *Γ*_*e*_^*+*^ = *Γ*_*e*_). Meanwhile, the decays of the resonance amplitudes are due entirely to the creation of the outgoing waves *s*_−_.

9 $$\frac{d({m}^{+}m)}{dt}=-{{s}_{-}}^{+}{s}_{-}=-{m}^{+}{D}^{+}Dm$$

Hence, we obtain *D*^*+*^*D* = 2*Γ*_*e*_ based on Eq. (8) and Eq. (9), which is a direct consequence of energy conservation. Since the coupled-SLSP system is symmetrical, it can be obtained:

10 $$\begin{array}{c}|{d}_{11}|=|{d}_{12}|=\sqrt{{\gamma }_{ea}}\\ |{d}_{21}|=|{d}_{22}|=\sqrt{{\gamma }_{eb}}\\ {e}^{(i{\varphi }_{12}-i{\varphi }_{11})}+{e}^{(i{\varphi }_{22}-i{\varphi }_{21})}=\frac{2{\gamma }_{c}}{\sqrt{{\gamma }_{ea}{\gamma }_{eb}}}\end{array}$$

Equation (10) indicates that the two modes generating the outgoing waves will decay into the two ports, maintaining a specific phase relationship. The designed coupling and excitation structure result in a π/2 relative phase difference between the antibonding and bonding modes (Fig. S1), yielding *φ*_12_ − *φ*_11_ = π/2 and *φ*_22_ – *φ*_21_ = π/2. Hence, we can obtain ${\gamma }_{c}=i\sqrt{{\gamma }_{ea}{\gamma }_{eb}}$ based on Eq. (9). Hence, the dynamic equations of the antibonding mode (*a*) and the bonding mode (*b*) are given by:

11 $$\frac{d}{dt}\left(\begin{array}{c}a\\ b\end{array}\right)=i\left(\begin{array}{cc}{\omega }_{a}+i{\gamma }_{a} & -\sqrt{{\gamma }_{ea}{\gamma }_{eb}}\\ \sqrt{{\gamma }_{ea}{\gamma }_{eb}} & {\omega }_{b}+i{\gamma }_{b}\end{array}\right)\left(\begin{array}{c}a\\ b\end{array}\right)+{K}^{T}\left(\begin{array}{c}{s}_{1+}\\ {s}_{2+}\end{array}\right)$$

According to Eq. (2), we can further calculate the transmission spectra of the coupled-SLSP system, which coincide well with the full-wave EM simulation results (Note S3).

## Supplementary information

Supplementary Information

## Data Availability

All data generated and analyzed during the current study are available from the corresponding author upon request.
