# Supplementary material for: Deep-subwavelength exceptional point in microwave plasmonic resonators for enhanced sensing
Source: Light Sci Appl. 2026 Aug 19;15:349. doi: 10.1038/s41377-026-02408-0 (PMC13490543; doi:10.1038/s41377-026-02408-0)
Supplement: Supplementary file 1 [file 41377_2026_2408_MOESM1_ESM.docx]

Supplementary Information for

**Deep-subwavelength exceptional point in microwave plasmonic resonators for enhanced sensing**

Tian Shuo Bai^1,2^, Xuanru Zhang^1,2,*^, Wan Zhu Wang^1,2^, Jingjing Zhang^1,2^, and Tie Jun Cui^1,2,*^

^1^State Key Laboratory of Millimeter Waves, Southeast University, Nanjing 210096, China

^2^School of Information Science and Engineering, Southeast University, Nanjing 210096, China

^*^Corresponding authors: zhangxru@seu.edu.cn, tjcui@seu.edu.cn

**Contents**

Figs. S1-S16

Notes S1-S5

Tables S1 and S2

**Fig. S1. Electric field distributions *E_z_* of the antibonding and bonding mode, the EP state, and the RP state vary with the transient phases.**

**Fig. S2. Simulated electric field distributions |*E*| along the radial direction (a) and the vertical direction (b) for the proposed EP sate, the spiral SLSP, and the conventional microstrip ring resonator (MRR).**

**Fig. S3.** **The simulated analysis of the structure-induced escaping decay for the single SLSP resonance unit.** **a** The structure of the single SLSP resonance unit with a certain rotation angle *α*. **b** The transmission spectra of the single SLSP resonance unit, varies as the rotation angle *α* increases. **c** The full width at half maximum (FWHM), which characterizes the mode decay coupling, varies as the rotation angle *α* increases. The intrinsic losses remain invariable, being solely attributed to the material absorption of the SLSP resonance unit.

**Fig. S4. The sensing signals and the average losses comparing the EP state and the *α* = 0° state as a function of perturbation strength in contactless scatterer detection.**

**Fig. S5. Simulated transmission (S_21_) spectra of the deep-subwavelength EP state in (a), spiral SLSP in (b), and conventional MRR in (c).** The diameter *D* of the lossy dielectric is set to 4 mm, with a thickness of 0.1 mm. To clearly highlight the sensitivity advantage of the deep-subwavelength EP state, the resonance frequency is normalized.

**Fig. S6. Photograph of the sensing experimental configuration and the fabricated EP sensor.**

**Fig. S7. Resonance frequencies of the EP sensor measured over an extended period.**

**Fig. S8. Measured resonance spectra of the EP sensor at 23 ℃ and 100 ℃.**

**Fig. S9. a** Photograph for multiple scatterers detection of the EP state. **b** Sensing performance of the EP state for multiple scatterer detection with different electrical sizes. **c** Sensing performance comparison between the EP state and the *α* = 0° state for single scatterer detection.

**Fig. S10.** **a** Frequency splitting of the *α* = 0° state responding to DI water, and the glucose of various concentrations. **b** Measured spectra of the *α* = 0° state for detecting glucose solutions of various concentrations with a VUT of 0.5 μL.

**Table S1.** Comparison with other reported non-Hermitian EP state.

|  | Frequency Range | Resonator Electric Size (λ_0_) | Normalized Frequency Detuning Δ*ω/ω*_0_ |
| --- | --- | --- | --- |
| Ref.^1^ | Optical | 50 ^a)^ | 1.20 × 10^−7 b)^ |
| Ref.^2^ | Optical | / | 1.69 × 10^−6 b)^ |
| Ref.^3^ | Microwave | 1/1.3 ^a)^ | 8.16 × 10^−4 b)^ |
| Ref.^4^ | Microwave | 1/2.7 ^a)^ | 1.90 × 10^−2 b)^ |
| Ref.^5^ | Microwave | 1/4.8 | 1.10 × 10^−2 b)^ |
| This work | Microwave | 1/50 | 3.00 × 10^−2^ |

^a)^ These numbers are calculated from other data reported in the literature.

^b)^ These numbers are calculated from figure curves reported in the literature.

**Table S2.** Comparison with other EP sensors for scatterer detection.

|  | **Resonator electric size** | **Resonator physical size** | **Quality factor** | **Principle** | **Detection limit** | **Δ*ω*/*ω*_0_** | **Contactless or not** | **Resonance**  **Frequency** |
| --- | --- | --- | --- | --- | --- | --- | --- | --- |
| Ref.^6^ | 50λ_0_ ^a)^ | 80 μm | 3 × 10^7^ | Extra scatterers | Not presented | / | Yes | Optical |
| Ref.^3^ | λ_0_/1.3 ^a)^ | 24 mm | 213 ^a)^ | Extra scatterers | Not presented | / | No | Microwave |
| Ref.^4^ | λ_0_/2.7 ^a)^ | 18 mm | 200 | Extra scatterers | λ_0_/100 | 0.004 ^a)^ | No | Microwave |
| Ref.^5^ | λ_0_/5 ^a)^ | 24 mm | 129 | Chiral resonator | λ_0_/280 | 0.006 ^a)^ | No | Microwave |
| Ref.^7^ | λ_0_/2.4 ^a)^ | 40 mm | 261 | Extra scatterers | λ_0_/1000 | 8.5 × 10^−4 a)^ | Yes | Microwave |
| This work | λ_0_/50 ^a)^ | 6.27 mm | 55 | Escaping decay engineering | λ_0_/1600 | 0.012 ^a)^ | Yes | Microwave |

^a)^ These numbers are calculated from other data reported in the literature.

**Note S1: Loss mechanism analysis of the antibonding and bonding modes**

The damping rate *γ_a_* (*γ_b_*) of the antibonding (bonding) mode is derived from the intrinsic loss and the escape decay from two physical ports for the *α* = 0° case, where there is weak intermodal coupling:

$$\left\{\begin{matrix} \gamma_{a}=\gamma_{0a}+\gamma_{ea}=\frac{1}{\tau_{0a}}+\frac{1}{\tau_{1a}}+\frac{1}{\tau_{2a}} \\ \gamma_{b}=\gamma_{0b}+\gamma_{eb}=\frac{1}{\tau_{0b}}+\frac{1}{\tau_{1b}}+\frac{1}{\tau_{2b}} \end{matrix}\right.$$

Here, *γ*_0_*_a_* = 1/*τ*_0_*_a_* (*γ*_0_*_b_* = 1/*τ*_0_*_b_*) represents the intrinsic loss of the antibonding (bonding) mode. *γ*_e_*_a_* = 1/*τ*_1_*_a_* + 1/*τ*_2_*_a_* and *γ*_e_*_b_* = 1/*τ*_1_*_b_* + 1/*τ*_2_*_b_* represent the escaping decay of the antibonding and bonding mode from two physical ports in the coupled-SLSP system. We remark that the intrinsic losses of the antibonding and bonding modes are equal (*γ*_0_*_a_* = *γ*_0_*_b_*) due to the mutual balance between material absorption loss and radiation loss, as shown in Fig. S11. Meanwhile, the radiation loss emitted into free space is minimal due to the deep-subwavelength confinement of the plasmonic resonance structure, and can be considered negligible.

**Fig. S1****1. Intrinsic loss analysis of the antibonding and bonding mode for the *α* = 0° case.** **a** Material absorption loss from the metal (copper) and the substrate (F4B). **b** Radiation loss emitted into free space.

Then, the multipeak fitting method in the main text is used to identify the aforementioned losses from the FWHM of the reflection and transmission spectra under different simulation settings, as shown in Fig. S12. The excitation via a single discrete port is weak enough to ensure minimal impact on the eigenmodes, making it suitable for analyzing the intrinsic losses of the antibonding mode and the bonding mode. The intrinsic losses of the antibonding mode and the bonding mode feature *γ*_0_*_a_* = *γ*_0_*_b_*, as shown in Fig. S12a. The escaping decays of the antibonding and bonding modes can be calculated in loss-free structure, assuming that their radiation losses are negligible due to the strong field confinement in deep-subwavelength resonance units. The escaping decays of the antibonding and bonding modes also feature 1/*τ*_1_*_a_* = 1/*τ*_1_*_b_*, as shown in Fig. S12b. In such a reciprocal two-port system, the escaping decays of the mode to port 1 and port 2 can be considered identical, with the relationships 1/*τ*_1_*_a_* = 1/*τ*_2_*_a_* and 1/*τ*_1_*_b_* = 1/*τ*_2_*_b_*. Hence, the above results indicate that the two split resonances feature approximately equal damping rates (*γ_a_* = *γ_b_*), as shown in Fig. 2e in the main text. Meanwhile, the damping rates *γ_+_* (*γ_−_*) of the mode + (*−*) can also be determined based on the loss fitting process described above before reaching the EP state (*α* > 0°), where the intermodal coupling is present.

**Fig. S12. Loss fitting process using the multipeak fitting method.** **a** Intrinsic loss 1/*τ*_0_*_a_* (1/*τ*_0_*_b_*) of the antibonding (bonding) mode for the *α* = 0° case. **b** Escaping loss 1/*τ*_1_*_a_* (1/*τ*_1_*_b_*) of the antibonding (bonding) mode from the waveguide port 1.

**Note S2: Impact of intrinsic losses on the coupled-SLSP system**

The regulatory capacity in coupling strength between the antibonding and bonding modes arises from the structure-induced escaping decay *γ_ea_* (*γ_eb_*) coupling to the ports rather than intrinsic losses of the modes *γ*_0_*_a_* (*γ*_0_*_b_*). Merely changing the intrinsic losses of the modes fails to achieve the expected EP state, as demonstrated in Fig. S13. The intrinsic losses of the modes are achieved by increasing the loss of the dielectric substrate (F4B). The transmission spectrum when the tangent of the loss is 0.001 is consistent with the *α* = 0° case in Fig. 2b of the main text. As the loss tangent of the dielectric substrate increases, the excitation efficiency of the transmission spectra decreases, accompanied by a broadening of the FWHM. However, the resonance peaks of the two modes remain fixed, demonstrating no tendency toward degeneracy. The dynamic process of the transmission spectra is completely opposite to Fig. 2b in the main text. This result also conforms to the structure-induced decay mechanism underlying the proposed deep-subwavelength EP state.

**Fig. S13. Impact of intrinsic losses on the coupled-SLSP system**.

**Note S3: Theoretical model based on temporal coupled-mode theory**

The coupled-SLSP system can be modeled using the temporal coupled-mode theory (TCMT) to analyze the coupling between the antibonding and bonding modes. According to the TCMT (see the derivation in the Materials and methods of the main text), the dynamic equations of the antibonding mode (*a*) and the bonding mode (*b*) are given by:

$$\frac{d}{dt}\left(\begin{matrix} a \\ b \end{matrix}\right)=i\left(\begin{matrix} \omega_{a}+i\gamma_{a} & \kappa_{ab} \\ \kappa_{ba} & \omega_{b}+i\gamma_{b} \end{matrix}\right)\left(\begin{matrix} a \\ b \end{matrix}\right)+K^{T}\left(\begin{matrix} s_{1+} \\ s_{2+} \end{matrix}\right)$$

The microstrip line on the backside carries a sinusoidal incoming wave *s*_1+_ towards the coupled- SLSP system. Port 2 remains without excitation (*s*_2+_ = 0) and hence *K*^T^ is degraded from 2 × 2 matrix to (*κ*_1_*_a_ κ*_1_*_b_*). The coefficient *κ*_1_*_a_* (*κ*_1_*_b_*) quantifies the degree of coupling between the antibonding (bonding) mode and the excitation *s*_1+_. The TCMT equations take the form:

$$i\left(\begin{matrix} \omega A \\ \omega B \end{matrix}\right)=i\left(\begin{matrix} \omega_{a}+i\gamma_{a} & -\sqrt{\gamma_{ea}\gamma_{eb}} \\ \sqrt{\gamma_{ea}\gamma_{eb}} & \omega_{b}+i\gamma_{b} \end{matrix}\right)\left(\begin{matrix} A \\ B \end{matrix}\right)+\left(\begin{matrix} \kappa_{1a}S_{1+} \\ \kappa_{1b}S_{1+} \end{matrix}\right)=i\hat{H}\left(\begin{matrix} A \\ B \end{matrix}\right)+\left(\begin{matrix} \kappa_{1a}S_{1+} \\ \kappa_{1b}S_{1+} \end{matrix}\right)$$

After substituting *κ_ab_* = −*κ_ba_* = $-\sqrt{\gamma_{ea}\gamma_{eb}}$, $a=Ae^{i\omega t}$, $\frac{da}{dt}=\frac{dA}{dt}e^{i\omega t}+i\omega Ae^{i\omega t}$(same for the bonding mode *b* and the sinusoidal incoming wave mode *s*_1+_) and $\frac{dA}{dt}=0$, $\frac{dB}{dt}=0$ in a steady state. Here, $\hat{H}$ represents the non-Hermitian Hamiltonian. Equation (3) is sorted into:

$$\left(\begin{matrix} i\text{Δ}\omega_{a}+\gamma_{a} & i\sqrt{\gamma_{ea}\gamma_{eb}} \\ -i\sqrt{\gamma_{ea}\gamma_{eb}} & i\text{Δ}\omega_{b}+\gamma_{b} \end{matrix}\right)\left(\begin{matrix} A \\ B \end{matrix}\right)=\left(\begin{matrix} \kappa_{1a}S_{1+} \\ \kappa_{1b}S_{1+} \end{matrix}\right)$$

where Δ*ω_a_* = (*ω* − *ω_a_*) and Δ*ω_b_* = (*ω* – *ω_b_*). Then, solving Equation (4) yields the amplitudes *A* and *B* in the following forms:

$$\left(\begin{matrix} A \\ B \end{matrix}\right)=\left(\begin{matrix} i\text{Δ}\omega_{a}+\gamma_{a} & i\sqrt{\gamma_{ea}\gamma_{eb}} \\ -i\sqrt{\gamma_{ea}\gamma_{eb}} & i\text{Δ}\omega_{b}+\gamma_{b} \end{matrix}\right)^{-1}\left(\begin{matrix} \kappa_{1a}S_{1+} \\ \kappa_{1b}S_{1+} \end{matrix}\right)$$

According to the relationship of energy conservation, the incoming and outgoing ports satisfy the following relationship:

$$S_{2-}=S_{1+}-\kappa_{1a}A-\kappa_{1b}B$$

Hence, according to equations (5) and (6), the transmission coefficients can be presented in the form:

$$T=\frac{S_{2-}}{S_{1+}}=1-\frac{\kappa_{1a}A+\kappa_{1b}B}{S_{1+}}=1-\left(\begin{matrix} \kappa_{1a} & \kappa_{1b} \end{matrix}\right)\left(\begin{matrix} i\text{Δ}\omega_{a}+\gamma_{a} & i\sqrt{\gamma_{ea}\gamma_{eb}} \\ -i\sqrt{\gamma_{ea}\gamma_{eb}} & i\text{Δ}\omega_{b}+\gamma_{b} \end{matrix}\right)^{-1}\left(\begin{matrix} \kappa_{1a} \\ \kappa_{1b} \end{matrix}\right)$$

The fitting results are shown in Fig. S14.

**Fig. S14. Theoretical analysis based on the TCMT model.** Evolution of the transmission spectra varying with the rotation angle *α*, calculated by the full-wave EM simulations and the TCMT model.

**Note S4: Near-field mapping system for** **verifying the fabricated EP sensor**

A self-made near-field mapping system is used to measure the |*E_z_*| fields for verifying the fabricated EP state, as shown in Fig. 4a of the main text. The sensor’s input and output ports are equipped with SMA connectors for exciting the EP state. The self-made near-field mapping system is composed of a scanner loaded with a near-field probe, displacement control system, and vector network analyzer (VNA). The input port of the vector network analyzer feeds energy into the EP sensor through the coaxial cable and one SMA connector, while the other SMA connector is connected to a 50 Ω matched load. The near-field probe is a vertical electric monopole, which was made by exposing the center conductor out of a coaxial cable. The near-field probe is positioned 4 mm away from the surface of the sensor. The distance is determined based on the simulated evanescent field characteristics of the EP sensor, as shown in Fig. S15. This ensures that the near-field probe causes minimal perturbation to the EM field of the EP state.

Then, the |*E_z_*| fields of the fabricated EP state at resonance frequencies were measured using the aforementioned self-made near-field mapping system. It can be observed that the electric field of one of the resonators is completely suppressed, while the electric field is localized in the other resonator. This is consistent with the simulated results shown in Fig. 4d of the main text and confirms that the fabricated sample is located indeed at the EP state.

**Fig. S15. Simulated evanescent field (|*E_z_*|) characteristics of the EP sensor along the *z* direction.**

**Note S5:** **Detection capability comparison**

Enhanced noise in the vicinity of EP states due to the nonorthogonality of the eigenmodes may result in a more conventional signal-to-noise ratio (SNR), compared to those sensors in a non-EP regime. This work demonstrates that the microwave sensors subjected to high noise can still achieve superior frequency shift detection capability, owing to high spectral resolution provided by microwave measuring equipment. The measured derivations (as shown in Fig. 4f in main text) are much smaller than the defined noise (averaged resonance widths). Schematic of the frequency shift detection capability under four conditions is demonstrated in Fig. S16, including (a) high resolution and narrow bandwidth, (b) high resolution and broad bandwidth, (c) low resolution and narrow bandwidth, (d) low resolution and broad bandwidth. The pixels denote the spectral resolution of microwave measuring equipment, while the blank pixels between the two resonance dips quantify the frequency shift detection capability. More pixels within the same frequency range correspond to a higher spectral resolution of microwave measuring equipment, and more blank pixels between the two resonance dips correspond to a greater frequency shift detection capability.

For high-resolution detection, sensors with different bandwidth exhibit identical frequency shift detection capability, as demonstrated in Figs. S16a and b. However, the frequency shift detection capability is reduced when the sensor features a broad bandwidth due to the low spectral resolution of the measuring equipment, whereas a sensor with a narrow bandwidth performs well, as demonstrated in Figs. S16c and d. In conclusion, the bandwidth is less critical when the measuring equipment possesses extremely high spectral resolution, such as a VNA for measuring microwave transmission and reflection spectra. Moreover, the impact of bandwidth cannot be ignored when the measuring equipment typically features lower spectral resolution.

**Fig. S16. Schematic of the frequency shift detection capability depending on the spectral resolution of microwave measuring equipment and the bandwidth of the sensors. a** High resolution and narrow bandwidth case. **b** High resolution and broad bandwidth case. **c** Low resolution and narrow bandwidth. **d** Low resolution and broad bandwidth case.

**References**

1. Peng, B. et al. Chiral modes and directional lasing at exceptional points. *Proceedings of the National Academy of Sciences of the United States of America* **113**, 6845-6850 (2016).
2. Hokmabadi, M. P., Schumer, A., Christodoulides, D. N., Khajavikhan, M. Non-Hermitian ring laser gyroscopes with enhanced Sagnac sensitivity. *Nature* **576**, 70-74 (2019).
3. Chen, J. et al. Coherent-resonance enhancement of sensing at the exceptional points. *Advanced Optical Materials* **12**, 2302268 (2024).
4. Liao, Z. et al. Microwave plasmonic exceptional points for enhanced sensing. *Laser & Photonics Reviews* **17**, 2300276 (2023).
5. Bai, T. S., Wang, W. Z., Zhang, X., Cui, T. J. Exceptional point in a microwave plasmonic dipole resonator for sub-microliter solution sensing. *Advanced Functional Materials* **34**, 2312170 (2024).
6. Chen, W., Özdemir, S. K., Zhao, G., Wiersig, J., Yang, L. Exceptional points enhance sensing in an optical microcavity. *Nature* **548**, 192-196 (2017).
7. Zhang, Y. et al. Reconfigurable exceptional point-based sensing with 0.001λ sensitivity using spoof localized surface plasmons. *Advanced Photonics Nexus* **3**, 056004 (2024).
